# The building blocks of social competence: Contributions of the Consortium of Individual Development

**DOI:** 10.1016/j.dcn.2020.100861

**Published:** 2020-09-18

**Authors:** Caroline Junge, Patti M. Valkenburg, Maja Deković, Susan Branje

**Affiliations:** a Departments of Developmental and Experimental Psychology, Utrecht University, Utrecht, the Netherlands; b Amsterdam School of Communication Research ASCoR, University of Amsterdam, Amsterdam, the Netherlands; c Department of Clinical Child and Family Studies, Utrecht University, Utrecht, the Netherlands; d Department of Youth and Family, Utrecht University, Utrecht, the Netherlands

**Keywords:** Social competence, Development, Model, Skills, Contexts, Cohorts

## Abstract

Social competence refers to the ability to engage in meaningful interactions with others. It is a crucial skill potentially malleable to interventions. Nevertheless, it remains difficult to select which children, which periods in a child’s life, and which underlying skills form optimal targets for interventions. Development of social competence is complex to characterize because (a) it is by nature context- dependent; (b) it is subserved by multiple relevant processes that develop at different times in a child’s life; and (c) over the years multiple, possibly conflicting, ways have been coined to index a child’s social competence. The current paper elaborates upon a theoretical model of social competence developed by Rose-Krasnor (Rose- Krasnor, 1997; Rose-Krasnor and Denham, 2009), and it makes concrete how underlying skills and the variety of contexts of social interaction are both relevant dimensions of social competence that might change over development. It then illustrates how the cohorts and work packages in the Consortium on Individual Development each provide empirical contributions necessary for testing this model on the development of social competence.

## 1 Introduction

Social competence can be characterized as the effectiveness of a child to engage in social interactions with peers and adults (Fabes et al., 2006; Rubin et al., 1998). It is the behavioral manifestation of a child’s emotional and regulatory competencies while interacting with other people. Social competence does not represent a fixed quality but should be viewed as a construct that in itself marks development: Society expects more sophisticated interactions with older children. When children are growing up, interaction contexts beyond the home environment gain importance and become increasingly broader. Moreover, being effective in a variety of social interactions requires children to master many skills that underlie social competence, such as perspective taking, social problem solving, and emotion regulation, which possibly also differ in developmental stadia. Knowledge about (a) these underlying skills, (b) the interaction contexts, and (c) these developmental stadia all contribute to a better understanding of social competence, which is why we consider these three types of knowledge as relevant dimensions, that is, as crucial building blocks of social competence.

Although research on social competence has made great progress in understanding underlying skills and relevant interaction contexts in key periods in children’s lives (see e.g., Rubin et al., 2009; Bukowski et al., 2018), how these building blocks of social competence connect to each other over the course of development is less well understood: still missing is a detailed model of the development of social competence from infancy to adolescence. The aim of the Consortium on Individual Differences (CID) is to contribute to such a model that captures the development of social competence in a changing society.

In what follows next, we first describe why the field is in need of a developmental model of social competence (Section 2). We then give a brief overview of the development of social competence from infancy to adolescence (Section 3). In Section 4, we explain the approach that CID takes towards building a developmental model, which is an elaboration upon a theoretical model of social competence developed by Rose‐Krasnor (1997; Rose-Krasnor and Denham, 2009). In Section 5, we show how each of the cohorts and the individual work packages from CID are contributing pieces of evidence to steer the theoretical model. Finally, in Section 6, we conclude by suggesting how the cohorts and work packages in CID can complement each other in building a developmental model.

## 2 Why it is crucial to have a better understanding of the development of social competence

Developing social competence is essential for future functioning in society and for reducing risk of behavioral and emotional problems. Indeed, there is ample evidence that variation in social competence in childhood is linked to prowess in other domains in present and later life. For instance, people who as children easily develop good relationships with others are more likely to grow into adults with better health (they live longer; are more resilient to mental health problems, and function better in society; Luthar, 2006; Masten and Coatsworth, 1995). Socially competent children are more likely to advance in academics (Caprara et al., 2000; Denham, 2006; Wentzel, 1991), or rate themselves as happier (Ryan and Deci, 2001). Reversely, deviances in social competence can be a symptom for many forms of psychopathology emerging in child development. If social competence appears deviant, many other problems are typically observed, such as peer rejection (in ADHD; Larson et al., 2011), social anxiety (La Greca and Lopez, 1998), bullying and aggression (Warden, and Mackinnon, 2003; for overviews, see Happé and Frith, 2014; Trentacosta and Fine, 2010). Together, this suggests that the construct of social competence is a key factor in explaining individual variation, both in typical and atypical child populations.

The construct of social competence is a developmental construct: it emerges from meaningful interactions with various others in a variety of contexts (Rose‐Krasnor, 1997). Such interactions shape children’s competence: children learn how to behave in their social worlds both through direct instruction as well as by observing others in interactions. As a result, the type and quality of interactions children experience become increasingly more varied and complex over time. Moreover, children’s concepts of the relevance of interactions mark clear progression. Clearly, the construct of social competence changes over time, but a unified model of how social competence emerges from infancy to adolescence remains missing.

There are several reasons why we need a better understanding how social competence unfolds. First, indexes of social competence from early childhood have been shown to be predictive of social competence later in life (e.g., Howes, 1987; Monahan and Steinberg, 2011; Rubin et al., 1998; but see Masten et al., 1995). In fact, there appears to be a Matthew effect for social competence: for example, those competent in making friends early in life are becoming more competent in forming friendships, while the less-competent ones are becoming even less competent in forming friendships (Flannery and Smith, 2017; Ladd, 1999; Monahan and Steinberg, 2011). Research further documents reciprocal links across various underlying skills of social competence. For example, positive experiences in building friendships early in life foster the development of prosocial behavior, which in turn increases the chance to form friendships later in life (Flannery and Smith, 2017; Ladd, 1999). Such self-reinforcing links between the underlying skills of social competence underscore the need to view the development of social competence as a dynamic, complex process in which children are actively regulating their own experiences and creating their own contexts (Sameroff, 2010). Yet to fully grasp the complexity of the development of social competence we need to better understand how and when social competence becomes self-reinforcing along development. Researchers should therefore start building and testing more elaborate models of social competence that take into account the interplay between development, the complexity of different underlying skills, and the variety of social contexts that together shape social competence.

A second reason why it is crucial to develop a clearer picture of how social competence unfolds is that social competence can be malleable, and open to interventions. Yet optimization of interventions in childhood requires not only identifying which underlying skills of social competence are well-suited targets, but also selecting optimal periods to administer such interventions, and should be tailored to a child’s stage of social competence. Knowledge on when to start an intervention is essential since developmental models such as the developmental cascades models assume that adaptive and maladaptive behaviors can result in spreading effects over time across various levels (Cicchetti, 2002). Optimal interventions should ideally result in the interruption of negative cascades and the promotion of positive cascades (Masten and Cicchetti, 2010). Thus, it is essential to develop a model of social competence that makes explicit not only how different underlying skills connect with different stages of social competence (the ‘hows’), but also how social competence changes over development (the ‘whens’).

The third and final reason why it is important to develop get a better picture on how social competence unfolds is that children’s social contexts (the ‘wheres’) have changed dramatically in the past two decades. One key change is that most Western infants and toddlers now have extensive experiences with peers and other adults prior to school entry. In fact, unlike earlier generations, most of today’s infants are in some form of day care away from their primary caregiver(s). How does this change affect the formation of peer relations and social competence (Hay et al., 2018)?

Another key change involves the rapid changes in children’s and adolescents’ media environments. In the 1970s the average age that a child started watching television was at 4 years of age. But due to the rise of prosocial and educational baby TV and apps (and parents’ tendencies to embrace such media), the onset of media exposure is now dropped to three and five months of age (Valkenburg and Piotrowski, 2017). Developmentally appropriate educational media may support cognitive learning (e.g., numeracy, literacy), but could also improve underlying skills of social competence (e.g., prosocial behaviour), particularly when adults are involved with the content their children consume (Courage and Howe, 2010). Furthermore, increasingly more interactions in childhood and adolescence take place online. What are the consequences of this? Do skills in social competence generalize easily to those required in online social interaction or does effectively communicating in digital interactions require an additional set of skills? Or does the larger amount of online interaction hamper development of complex underlying skills of social competence, such as emotion recognition and perspective taking? This is something research only starts exploring (Blumberg et al., 2019).

## 3 Sketching the development of social competence

Before we can explain how CID aims to build theory on the development of social competence, it is essential to provide an overview of how social competence develops across childhood. In Table 1 we therefore define each period in childhood and list the main characteristics in marking the development of social competence. Please note that this overview is neither inclusive nor complete—it only serves to outline the highlights of each period in relation to social competence.

**Table 1:** Each age period comes with its own characteristics of social competence.

Developmental period	Highlights
Infancy (0−1 years)	- First prominent social context is with primary caregiver(s) - Vital markers to SC evident from parent-infant interactions (attachment, parental responsiveness) - Marks temperament as a biological trait - Onset of social responses (smiling, vocalization, pointing, imitations of facial expressions) - Emergence of awareness to social stimuli (facial emotions, word comprehension; social referencing)
Early Childhood (2−5 years)	- Social interactions become more varied and complex - Rudimentary beginnings of perspective-taking skills - Prosocial behavior emerges (sharing and helping) - Play in dyads with age-mates, but under control by parents - Play progresses from parallel play to social play - Sensitivity to positive peer status (prosocial behaviour, cooperation and fairness)
Middle Childhood (6–12 years)	- School becomes a dominant social context, making evident social dominance hierarchies (sensitivity to peer popularity) - Friendships center on peer acceptance - Sensitivity to aspects of poor social competence (withdrawal, verbal aggression and defiance) - Perceptive taking skills further develops by taking into account other’s perspective in social situations (focus on gaining peer acceptance and avoiding peer rejection)
Adolescence (12–18 years)	- Social interactions less tied to school - Friendships center on intimacy and reciprocity - Interactions take place with peers in social cliques - Development of identity (mainly based on one’s cliques) - Perspective skills matures as awareness grows that others have different needs - Beginnings of romantic relationships

Certainly not surprising, it appears that any period in a child’s life is fundamental in contributing to social competence (Rubin et al., 2009), albeit for different reasons. For example, while in infancy social interaction skills typically evolve within the family context (e.g., Jones et al., 2014), childhood highlights the dominating force of peers within the classroom (Masten and Coatsworth, 1995), and adolescence is the period in which most relevant social interactions mainly take place in cliques (Moffitt, 1993; Weiss, 1986). In addition, a skill such as perspective taking emerges in early childhood but only reaches mature levels in adolescence, when adolescents have learned to appreciate that others can have different opinions (Selman, 1980). Although each period comes with its own developmental tasks, most central issues continue to be of importance throughout development (Waters and Sroufe, 1983). For instance, the significant association between the quality of parent-child relationship and children’s social competence is not moderated by age (Groh et al., 2014). A developmental model of social competence should thus not only view its development as a set of discrete stages, but also consider the factors that continue to bear on its development.

## 4 Towards a developmental model of social competence

How should we now start building a developmental model of social competence? We propose to build on an existing theoretical model: the prism model of social competence put forward by Linda Rose‐Krasnor (1997; Rose-Krasnor and Denham, 2009). This model does not focus on the development of social competence, but describes the different elements required for establishing good social interaction. We will first briefly summarize the prism model, before we outline how CID makes the prism model more concrete by adding a developmental framework.

The prism model has three hierarchical layers of analysis of social competence and one depth- dimension (context). The top layer of analysis is the theoretical one, which concerns social competence defined as effectiveness in interaction (Rose‐Krasnor, 1997). This definition allows us to maintain the same definition from infancy to adolescence. The second layer contains the indexical level and relates to the various ways in which social competence can be measured (Flannery and Smith, 2017). The bottom layer of the prism model is the skills- dimension, which lists those underlying skills that are important across the many different contexts in which social interactions take place, such as emotion regulation and perspective taking skills. Finally, the depth- dimension of the prism model reflects the various kinds of contexts (home vs school; parent vs. peers; online vs. offline) in which interaction takes place.

**Fig. 1:**
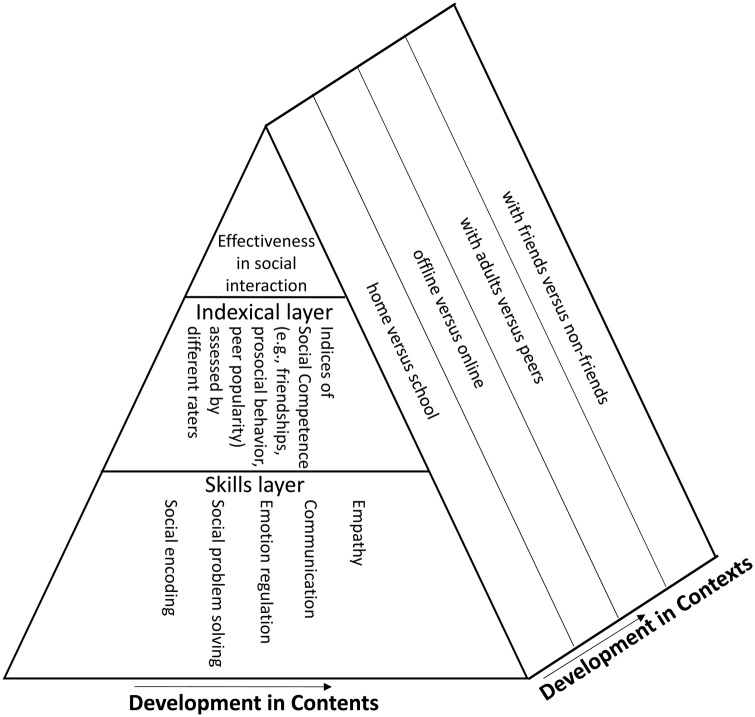
CID’s adaptation from Rose-Krasnor’s model of social competence (Rose‐Krasnor, 1997; Rose-Krasnor and Denham, 2009), adding a developmental perspective.

In the next sessions, we explain how CID implements and provides data for the indexical layer as well as the skills- and context-dimensions in more detail. See Fig. 1 for a schematic representation of our proposed model based on Rose‐Krasnor (1997; Rose-Krasnor and Denham, 2009).

### 4.1 The indexical layer

The indexical layer encompasses the numerous ways researchers employ to quantify social competence, each of which characterize aspects of social competence or underlying skills of social competence (cf., Fabes et al., 2009; Flannery and Smith, 2017). The cohort studies in CID mainly rely on questionnaires as these are one of the easiest, fastest and most common ways to collect information about social competence in large groups of children (El Mallah, 2020; Halle and Darling-Churchill, 2016). Most questionnaires are standardized, normed and internationally known questionnaires that can be filled in by either parents, teachers or children themselves.

### 4.2 The skills-dimension

The skills dimension is concerned with the foundational skills and motivations underlying social competence that are primarily individual in nature. It is at the skills level that developmental change might be considered most prominent and open to interventions (Rose‐Krasnor, 1997). However, there is no consensus on what one considers vital skills, partly because it is often difficult to tease apart underlying crucial skills from manifestations of social competence itself. Take for instance social perspective taking, which can be viewed both as an index of social competence, as well as a necessary skill from which social competence thrives. Table 2 lists the skills that various researchers find crucial for social competence (Crick and Dodge, 1994; Halberstadt et al., 2001; Hay et al., 2004; Raver and Zigler, 1997; Rose‐Krasnor, 1997; Rose-Krasnor and Denham, 2009). Although this list should not be considered as complete, it shows the variety of skills involved in social competence.

**Table 2:** An overview of studies that list various skills as relevant processes to social competence.

	Skills relevant to social competence
Crick and Dodge, 1994	1 Encoding social situation 2 Interpreting social situation 3 Arousal regulation 4 Response construction 5 Response evaluation and selection 6 Behavioral enactment
Halberstadt et al., 2001	1 Awareness 2 Identification 3 Working within social context 4 Management and regulation
Hay et al., 2004	1 Joint attention 2 Emotional regulation 3 Inhibitory control 4 Imitation 5 Causal understanding 6 Language
Raver and Zigler, 1997	1 Emotion regulatory skills 2 Social cognition skills 3 Thoughts, beliefs and attitudes about relationships; 4 Emotion labels; 5 how children feel about themselves. 6 Communicative behaviors (both verbal and nonverbal)
Rose‐Krasnor, 1997	1 Perspective taking 2 Communication 3 Empathy 4 Affect regulation 5 Social problem solving
Rose-Krasnor and Denham, 2009	1 Self-regulation 2 Social-problem solving 3 Prosocial behavior 4 Social awareness 5 Communication abilities 6 Sociodramatic play
Junge et al. (this paper)	1 Social encoding 2 Social problem solving 3 Emotion regulation 4 Communication 5 Empathy

Crucially, while Table 2 serves to highlight that there is no consensus in what one considers vital skills for social competence, it also reveals points of intersection. By focusing on those skills that are repeatedly listed we assume that these skills reflect the key foundations for social competence. We selected a set of five skills that serve as possible indicators in representing children’s (potential for) social competence. Below we motivate our choice in more detail. We begin with providing a definition and signaling its agreement with other researchers from Table 2. We then give a brief overview of development, and end with how interventions targeted to this skill are beneficial for social competence.

#### 4.2.1 Social encoding

Social encoding is the skill that requires a child to attend to the social interaction partner and to interpret meaningful cues from this person, such as emotions. We see the relevance of social encoding to social competence also in other researchers’ inventories of necessary skills (albeit phrased somewhat differently): as ‘encoding social situations’ (Crick and Dodge, 1994), as ‘awareness and identification’ (Halberstadt et al., 2001) and as ‘joint attention’ (Hay et al., 2004). Some researchers suggest that newborns’ early interest in faces may be ‘the gateway to social expertise’ (Jones et al., 2014). There is evidence that already seven-month- olds can differentiate between facial expressions (Leppänen and Nelson, 2009), although the decoding of human faces continues to develop into adolescence (e.g., Cohen Kadosh et al., 2013; cf. Blakemore, 2008). Our proposal that social encoding is one of the key foundations of social competence is supported by interventions demonstrating that social encoding lead to modest improvements in children’s social competence (Trentacosta and Fine, 2010).

#### 4.2.2 Social problem solving

Social problem solving (Rose‐Krasnor, 1997; Rose-Krasnor and Denham, 2009) can be considered a logical continuation of the previous skill (social encoding), as it centers on responding in such a way to achieve social goals, such as solving conflicts with peers or gaining access to peer play. This skill is also listed by some as ‘social decision making’ (Crick and Dodge, 1994). From early childhood up to adolescence, as children function increasingly in groups, social decision making assumes importance and often revolve around conflict resolution. One way to end conflicts is to react with anger or aggression, which often links to negative outcomes of social competence such as peer rejection (Card and Little, 2006; Von Salisch and Zeman, 2018; Werner and Crick, 1999). This is not only true for behavior at the playground, but also holds for on-line behavior: cyber aggression is related to higher rates of loneliness and lower rates of friendships (Schoffstall and Cohen, 2011). There are developmental shifts in the type of aggression that children can show in conflicts (Laursen and Pursell, 2009), and when children use aggression strategically, it might actually be considered beneficial (Hawley et al., 2007). Like social encoding, social problem solving is a skill susceptible to interventions aimed at improving social competence (Denham and Almeida, 1987; for a recent meta-analysis, see Merrill et al., 2017).

#### 4.2.3 Emotion regulation

If there is one skill that all researchers included in Table 2 consider vital to social competence, it is emotion regulation (Hay et al., 2004; Raver and Zigler, 1997; also referred to as ‘arousal regulation’; Crick and Dodge, 1994; as ‘affect regulation’; Rose‐Krasnor, 1997; or as ‘self-regulation’; Rose-Krasnor and Denham, 2009; Vink et al., 2020). Being unable to exert control over one’s emotions, behaviors and arousals while interacting with others is a clear sign of obtrusive, unpleasant behavior that is typically disliked by most people. Indeed, there is ample evidence linking poor regulation skills to negative indices of social competence, in particular peer problems (e.g., Eisenberg et al., 2001; Holmes et al., 2016; cf. Eisenberg et al., 2010). As inter alia Vink and colleagues describe (Vink et al., 2020), emotion regulation is an umbrella term that covers both effortful control as well as executive functions and executive control (see also Nigg, 2017). In early infancy, children’s responses are at first mainly reactive rather than pro-active (Ruff and Rothbart, 1996). Processes related to executive functions also come to the scene, mainly in toddlerhood onwards; for instance, inhibitory control emerges around 24–26 months (Kochanska et al., 2000), whereas improvements in executive control appear most pronounced in early childhood (Carlson, 2005). A recent review on the development of emotion regulation (Eisenberg et al., 2010) reveals that children make great advances in their ability to exert control over their emotions in the preschool years while it improves more slowly into adulthood. Importantly, while individual differences in emotion regulatory skills are rather stable, they can serve as a mediator between parenting and children’s problem behaviors (Belsky et al., 2007; Van Dijk et al., 2017). Moreover, interventions targeted at promoting self-regulation or regulating emotions result in more socially competent students (Domitrovich et al., 2007; Low et al., 2015).

#### 4.2.4 Communication

Communicative competence refers to the ability to use language effectively and appropriately in different social situations (Hymes, 1979). Developing good communication skills (Raver and Zigler, 1997; Rose‐Krasnor, 1997; Rose-Krasnor and Denham, 2009) is of course also essential in ‘competent responding’ required for sustaining positive engagement in interactions (Crick and Dodge, 1994). Although communication involves also nonverbal understanding (Raver and Zigler, 1997), it is language that is indispensable to communication. Although language shows marked improvements in all aspects from infancy till early childhood (e.g., Clark, 2003), it is in particular a child’s pragmatic abilities (e.g., concerned with how children use language in interactions) that prove most relevant to social competence. For instance, children who scored low in pragmatic abilities (e.g., offered fewer requests for explanations or clarifications, initiated fewer conversations, and showed inappropriate turn-taking behaviors) were more likely to be rejected by their peers (van der Wilt et al., 2018). In addition, children with developmental language disorder often experience peer problems or display problem behaviors (e.g., Curtis et al., 2018; Forrest et al., 2018; Van den Bedem et al., 2018), but this is related to pragmatic rather than structural problems with language (St. Clair et al., 2011; Van den Bedem et al., 2019). Interventions aimed at improving pragmatic skills prove beneficial in promoting social competence and reducing peer problems (Adams et al., 2012; Bierman et al., 2013; Coplan and Weeks, 2009).

#### 4.2.5 Empathy

Empathy is a broad concept which generally entails the skill of identifying with another by taking another person’s perspective (cognitive empathy) as well as sharing the emotions of others (affective empathy). Empathy thus acknowledges the awareness that other people may have different emotions and feelings, but also allows for responding appreciatively, both of which are important prerequisites for maintaining social interactions (Eisenberg Fabes, & Spinrad, 2006). Because empathy is a highly valued trait in interactions, it is relevant for a myriad of social competence indices such as sustaining relationships, forming friendships, and peer popularity (Eisenberg et al., 2015, 2006; Spinrad and Gal, 2018).

Both affective empathy (responding to other person’s emotions, for instance via imitation) and cognitive empathy (‘social perspective taking’; Rose‐Krasnor, 1997) are considered vital skills for social competence (Rose‐Krasnor, 1997). We see the relevance of empathy to social competence also acknowledged by other researchers in Table 2: Hay and colleagues list it as ‘imitation’ as well as ‘causal understanding’ (Hay et al., 2004); Rose‐Krasnor (1997) as ‘empathy’ and ‘perspective taking’.

It is possible that affective and cognitive empathy have different developmental paths. For affective empathy, it appears that even neonates can already imitate other’s facial expression (e.g., contagious crying, Simner, 1971). There is evidence that affective empathy in childhood and adolescence is an important underlying skill of social competence (e.g., Van der Graaff et al., 2014; van Hoorn et al., 2016). There is also protracted development in social perspective taking, which starts at a later age (Rose‐Krasnor, 1997; Selman, 1980). Toddlers begin social perspective taking by recognizing the separation between self and others. That is, they are developing a theory mind, which is the awareness that others can hold different feelings or opinions from themselves (Wellman, 1992; Wellman et al., 2011). Across childhood (2–12 years) children who possess an advanced theory of mind often display higher levels of social competence (Imuta et al., 2016). Yet preschoolers might still find it difficult to act upon it as their own feelings might be a more dominating force. It is only by late childhood that children learn to view oneself from another person’s perspective. Early adolescence sees the development of mutual and third- person perspective, and late adolescence is characterized by taking into account perspectives beyond the immediate interaction as it considers the relevance of one’s current interaction to social norms.

Interventions targeting the skill of empathy often start with improving in social-emotional understanding and prosocial behaviors in class-room settings (that is, in early and late childhood). Such interventions reveal small but positive effects for fostering social competence, visible in indices such as peer nominations and teacher ratings (Durlak et al., 2011; Malti et al., 2016).

To conclude, for the skills-dimension CID identifies five skills underlying social competence, each of which are complex constructs of themselves.

### 4.3 The context-dimension

The context dimension stresses the variety of relevant contexts in which interactions usually can take place in Western society. Such contexts do not only concern the setting of an interaction (“a situation in time and place”; Hartup, 2009, p.8), but also with whom a child is interacting. While researchers acknowledge the variety in the skills contributing to social competence, few make explicit the variety of contexts that shape social competence. To demonstrate the richness of contexts of these interactions it is helpful to characterize them using pairs of dichotomies. Below we give four useful dichotomies, and sketch development.

#### 4.3.1 Home versus school

It is at home that children will build the first set of meaningful interactions, with their caretaker(s) and with the other members of the household (e.g., siblings). Consequently, the home provides the foundation for social interactions. Nevertheless, this is not the only context in which some infants learn to interact with others. In Western societies such as the Netherlands, the majority of infants and toddlers regularly experience a form of daycare or play groups, which provides opportunities to learn to interact within small and stable groups of age mates (Hay et al., 2018). Most people agree that providing such additional contexts can be beneficial for a child’s development of social competence, but how or when to cater for this is poorly understood. Next, while infants and toddlers differ in how much of the home context provides the dominant social context relative to other contexts, it is in early and late childhood that for all children the classroom setting gradually becomes a dominant social context. This is why indices on social competence collected in childhood often revolve around group dynamics in the classroom setting, such as (perceived) peer popularity and peer rejection (Asher and McDonald, 2009). In adolescence, the major social context is still dominated by peers, but this time from their own cliques and clubs rather than the classroom.

#### 4.3.2 Offline versus online

Early in infancy most of the interactions take place offline, in close proximity of other persons. With the rise of social media, children come into contact with multiple forms of online interactions from an early age. Indeed, there is evidence that even infants can also learn from persons via on-line interactions, as long as there is social contingency between the child and the other (i.e., when turn-taking occurs naturally, and not artificially; Roseberry et al., 2014). Children in middle childhood and adolescents increasingly use social media tools to communicate or play with their friends, peers, or partners they meet on game or other types of platforms (Valkenburg and Piotrowski, 2017). A recent survey from the Netherlands has shown the majority of adolescents use two or three social media platforms, such as Instagram, YouTube, and Snapchat, in a complementary way (van Driel et al., 2019v). Today’s adolescents are amongst the first cohorts of young individuals who have grown up using mobile devices and social media; unlimited access to digital technologies enables them to be in constant contact with their peers and to engage in various social activities, such as playing games, creating audiovisual content, and sharing knowledge (Salmela-Aro et al., 2017). However, about ten percent of current social media users has been identified as compulsive social media users (van den Eijnden et al., 2016v). Furthermore, positive correlations between compulsive use of technology and comorbid psychiatric disorders have been reported (Andreassen, 2015). Since social media are a relatively new phenomenon, many questions regarding their potential impact on social competence and mental health remain unanswered (Pantic, 2014). Therefore, more research in this field is required, and we hope that CID may provide some initial answers.

#### 4.3.3 With adults versus peers

In a child’s life the most important adults are the caretakers (usually, the parents), and from childhood onwards, the teachers as well. (In both cases the child typically cannot control these social interaction partners). There is ample evidence that both parents’ (Feldman et al., 2013; Groh et al., 2014) as well as teachers’ characteristics (Wentzel, 2009) provide opportunities of interactions that contribute to a child’s social competence. From early childhood onwards, age-matched peers become increasingly the favored choice of interaction partners, as children learn to play and interact with peers. Although children with good social competence can interact easily in both contexts, children with poorer social competence (e.g., shy, withdrawn) find it often easier to interact with adults than with age-matched peers. Therefore, whereas adults might judge a child to be socially competent, one might reach different conclusions when observing a child interacting in situations with other peers.

#### 4.3.4 With friends versus nonfriends

Already preschoolers can distinguish between friends and nonfriends (Howes, 2009). Friendships centers around concepts of similarity: children like to play with others who are like themselves. However, our definition of social competence also requires that good social competence skills may come to the surface in contexts when the interaction partner is not familiar to the child. That is, how does the child interact when the other is not a friend, and the child therefore may not feel at ease with? Children who are shy in talking to others, or even experience social phobia, are at increased risk of developing poor social competence. It is therefore also important to consider social competence in the context of interaction partners the child is not friends with (Asher and McDonald, 2009).

## 5 CID contributions to the developmental model for social competence

In the current paper we set out explaining that one of the aims of CID is to grasp the development of social competence. CID is a consortium of Dutch researchers aiming to understand the extent and relevance of individual differences in development. There are four main themes of research in CID, grouped into 4 work packages accordingly. Each work package focuses on different aspects of explaining individual differences across development. Two longitudinal cohorts were set up: the YOUth cohorts to sample neurocognitive development (Work Package 1;Onland- Moret et al., 2020), and the Leiden-CID cohorts (‘L-CID’) to test interventions in twins (Work Package 2; Crone et al., 2020). Work Package 3 unites four current cohorts established prior to CID (Branje et al., 2020): Generation R (‘Gen-R’, Kooijman et al., 2016); Netherlands Twin Register (‘NTR’, Boomsma et al., 2006); RADAR (e.g., Branje and Meeus, 2018; Crocetti et al., 2017) and TRAILS (Ormel et al., 2012). Finally, Work Package 4 focuses on advanced statistical modelling and animal models. CID thus encompasses six Dutch large-scale longitudinal cohort studies capturing child development through repeated measurements while it also houses the tools and methods required to address the complexity in developmental research. In what follows next, we provide more information how each of the cohorts (Section 5.1) and work packages (Section 5.2) provide building blocks towards building this model.

### 5.1 Contributions from the cohorts in CID

The cohorts participating in CID aim to help building a developmental model of social competence that integrates the ‘whens’ (age periods), ‘whats’ (indexical layer), ‘hows’ (skills-dimension) and ‘wheres’ (context- dimension) of social competence (See Fig. 1). More specifically, all cohorts in CID provide information about the ‘whens’ and ‘whats’ as all sample the development of social competence, albeit they differ in how exactly. It is one of the strengths of CID that all cohorts employ multiple indices collected at various moments in a child’s life to capture a child’s current stage of social competence. Table 3 lists for all cohorts which questionnaires they include to index social competence and it provides information about the age ranges that are covered; the frequency of administration, and the respondent filling in the questionnaires (amongst others children, parents, teachers).

**Table 3:** An overview of the questionnaires that tap social competence and skills underlying social competence, for each of the cohorts involved in CID, with ages in years sampled in brackets.

Social Competence Indices	CID COHORTS					
*Cohorts:*	**Gen R**	**L-CID**	**NTR**	**RADAR**	**TRAILS**	**YOUth**
ASQ-SE-2				X^p^ (1−5) ^2^	X ^p^ (2.5)	X ^p^ (0.5; 0.10; 2−5)
CBCL	X ^p^ (1.5 ;3; 6; 10;14)		X ^p^ (7; 9/10; 12) X ^t^ (7; 9/10;12) X ^c^ (14;16;18)	X^p^ (2−5) ^2^ X^t^ (5)	X ^p^ (2.5; 4.5) X^t^ (4.5) X^p^ (11−16)^3^X ^t^(11−16) ^3^X ^c^(11−16) ^3^	X ^p^(2–7; 8 −16)^3^
IRI		X *^c^* (11; 12) ^1^		X ^c^ (12−27)^1/2^		X ^p^ (2−7)^3^X ^c^(8 −16)^3^
ITSEA				X ^p^ (2−5)^2^	X ^p^ (2.5)	X ^p^ (2−5)
NRI				X ^p^ (12−18)^1^X ^f^(12−18) ^1^X ^s^(12−18) ^1^X ^i^(18−27) ^2^X ^c^(12−27) ^1,2^		X ^p^(8−16) ^3^ X ^c^(8 −16)^3^
Prosocial subscale*				X ^c^ (12−27)^1, 2^	X ^t^(11 −16)^3^	
SDQ	X **^p^ (6; 10)	X ^p^ (3−9; 7–13)^1^X ^c^(11 –13) ^1^	X ^p^ (9)	X ^p^ (2−5) ^2^	X ^p^ (4.5) X ^t^ (4.5)	X ^p^ (2−7; 8−16)^3^X ^c^(11 −16)^3^
SSRS			X ^t^ (7; 9/10;12)		X ^p^; (11) X^t^ (11)	

Note:

*Cohorts*: Gen R = Generation R; l-CID = leiden Consortium on Individual Development; NTR = Netherlands Twin Register; RADAR = Research on Adolescent Development and Relationships; TRAILS = Tracking Adolescents’ Individual Lives’ Survey; YOU-th = Youth of Utrecht.

*Indices*: ASQ = Ages & Stages Questionnaire – Social Emotional -2 (Squires et al., 2002); CBCL: = Child Behavior CheckList (Achenbach, 1991; Achenbach and Rescorla, 2001); IRI = Interpersonal Reactivity Index (Davis, 1983); ITSEA = Infant-Toddler Social and Emotional Assessment (Briggs-Gowan et al., 2004); NRI = Network Relationships Inventory (Furman and Buhrmester, 1985); SDQ = Strengths and Difficulties Questionnaire (Goodman, 1997, 2001); SSRS = Social Skills Rating System (Gresham and Elliott, 1990).

* Prosocial subscale from the Revised Self-Report of Aggression and Social Behavior Measure (Morales and Crick, 1998).

** – only the prosocial scale from the SDQ.

^c^ self-report; ^p^ parent report; ^t^ teacher report; ^f^ friend report; ^s^ sibling report; ^i^ partner report.

1 = collected every year; 2 = collected every two years; 3 = collected every three years.

Indeed, one of the strengths of CID is that its variety in questionnaires allows us to sample every period of child development, starting with infancy (YOUth cohort) and toddlerhood (L-CID cohort). This is in contrast with most studies that only begin measuring social competence once children go to school (Parker et al., 2006). Because the CID cohorts cover each period in child development, we can examine not only direct and long-term outcome measures of social competence, but also precursors to social competence in younger children.

Another strength of CID is that these questionnaires are filled in by a variety of raters (children themselves, parents, teachers, or others), as the source of ratings might be prone to rater bias (Jones and Yudron, 2016). It is important to consider the source of ratings (for example, parent-report vs. self-report), as the source often makes a difference on the factor loadings of the assumed underlying construct (e.g., Goodman, 2001; Van Roy et al., 2008).

Although the cohort studies in CID use a variety of indices of social competence, two are used in virtually all cohorts: the Strengths and Difficulties questionnaire (SDQ: Goodman, 1997; for Dutch: Van Widenfelt et al., 2003) and the Child Behavior Checklist (ASEBA CBCL; Achenbach, 1991; Achenbach and Rescorla, 2001; for Dutch: Verhulst et al., 1996). Both questionnaires are relevant indices of social competence; they measure different underlying skills and complement each other. Whereas the SDQ measures key underlying skills such as prosocial behavior and friendship behaviors, the CBCL focuses on atypical (problematic) behavior in social interactions. Because all our cohorts employ at least one of these two questionnaires, CID is eventually able to collapse indices of social competence across cohorts that share the same indices (see also Zondervan-Zwijnenburg et al., 2020 for a similar approach to combining multiple cohort data on questionnaires related to behavioral control).

Besides the indexical layer, the cohorts participating in CID also address each of the five identified skills from the skills dimension, via repeated measurements collected in multiple ways spanning development from infancy into adulthood: through questionnaires, experimentally, and in parent-child interaction tasks. In Table 1 from the supplementary information we further delineate how each of the CID cohorts captures these five skills we consider relevant building blocks of social competence. Other articles in this special issue discuss some of the tasks and questionnaires in more detail.

With information adding to the development of skills we can ultimately understand the interplay between different indices, different subserving skills, and different contexts. This is crucial as social competence is a complex developmental construct. Take for instance the development of the underlying skills. While each of these skills show development, they often differ in their trajectories, and operate at different time scales at which they are more influential for social competence than others (e.g., Happé and Frith, 2014). We therefore do not assume that development in each skill proportionally continues to shape the development of social competence but rather that weights will change over time.

We illustrate the different time courses by comparing the communication versus empathy skills. Each of these skills have shown to be crucial, but how do they compare to each other in their relevance to social competence? Communication requires in particular a good command of pragmatics in order to confer meaning appropriately for social interactions. For communication skills we assume that pragmatic development has profound influences on social competence in childhood (van der Wilt et al., 2019), but that the additional relevance of language development for social competence might reach a plateau over the following years, before it again assumes importance when friendships in adolescence center on intimacy & self-disclosure (Troesch et al., 2016, but see Curtis et al., 2018). Nevertheless, our changing society might also add further relevance to communication skills, as interactions increasingly take place online. It is unclear for instance how children with or without developmental language delays fare in digital media contexts that does not require immediate responses (Drago, 2015).

In contrast to the relevance of communication skills to social competence in the early years, cognitive empathy is a skill that shows marked development in the adolescent years (van der Graaff et al., 2014). We therefore expect this skill to continue to grow in importance to social competence, possibly peaking in adolescence, as this is the period when social perspective taking becomes sophisticated (Selman, 1980) and when peer influence becomes a major force in social decision making (e.g., Crone and Dahl, 2012).

The above illustrations are mainly speculations. With the evidence gathered so far, we can only isolate the time course of the skills to underlying social competence and provide estimates how their relevance changes over time. What is still missing is evidence that reveals how a range of underlying skills across development together shape the development of social competence. Moreover, given that there is development both in the skills underlying social competence as well as in the different aspects characterizing social competence, such data will also unravel whether there are bidirectional relationships between skills and outcome measures. To illustrate, a recent study shows that while empathy predicts development in friendship quality, the reverse also holds: friendship quality drives empathy development (Van den Bedem et al., 2019). Because CID repeatedly collects information on a wide range of skills (Table 1 from S.I.) concerning the same children from whom we also collect indices of their social competence (Table 3), we aim to eventually contribute the evidence required for a better understanding how these skills work in tandem towards the development of social competence.

### 5.2 Contributions from the work packages in CID

Above we listed how the cohorts within CID examine the building blocks of social competence, as we make concrete how social competence emerges out of a variety in skills and contexts. Even so, fully capturing (the range in) the development of social competence requires integrating biological, psychological, and environmental factors, as well as insights into how these processes influence one another over time (Beauchamp and Anderson, 2010; Karmiloff-Smith, 2017; Karmiloff-Smith et al., 2014). Further, in-depth understanding of individual differences in social competence begs a more detailed understanding of each of the descriptive levels of analysis, ranging from the molecular to the behavioral level, and how these levels link to each other both at the same time and across development. However, to date it has been virtually impossible to predict which combinations of factors at which times explain individual variability in the development of social competence.

One of the main reasons why there is not yet such a detailed account is that while different strands of research provide relevant blocks of knowledge, these remain limited as they typically do not cross beyond the boundaries of their own scholarly discipline. To illustrate, developmental studies often rely on longitudinal studies to investigate how psychological child characteristics and environmental factors contribute to a child’s well-being in real-life (Bronfenbrenner, 2005), but these studies often do not include a biological or neurocognitive levels through which factors affect social competence (but see Crone et al., 2020). In contrast, biologically-oriented models provide us with a detailed mechanistic understanding of genes, neural function, or brain maturation relevant to the development of social cognition (e.g., Bakermans‐Kranenburg and Van IJzendoorn, 2007; Blakemore, 2008; Happé and Frith, 2014; Johnson et al., 2015; Robinson et al., 2008; Werker and Hensch, 2015) but they do not take into account child characteristics such as emotion regulation. It is here that the CID proves instrumental to building a developmental model of social competence as it accommodates the various disciplines of research that examine the development of social competence in both online and offline interactions as well as possess the statistical knowledge to integrate these findings.

As noted above, social competence is a developmental outcome measure that is reciprocal in the long-term. This makes social competence an example of a developmental cascade as it reflects behavior that can prove adaptive for some while having maladaptive consequences for others. Masten and Cicchetti (2010) identify five strands of research that would inform and optimize interventions required to promote positive cascades but to interrupt negative cascades: all of which are available in the work packages in CID.

One of the proposed strands is that research should determine when the cascade of social competence begins and accelerates to optimize the timing of interventions (Masten et al., 2009). As laid out in this paper, data collected in our work packages 1–3 together provides an overview of social competence spanning from 20 weeks’ pregnancy (YOUth cohort) to far into adulthood (e.g., RADAR, TRAILS). Consequently, we cover development of social competence completely; that is, we can observe precursors in pregnancy, infancy and toddlerhood as well as its long-term consequences from conception and infancy onwards.

Second, cascade models would benefit from repeated measurements of social competence collected at various overlapping time scales. The choice of a lag is often chosen arbitrarily, while there must be adequate time for the cascading effects of factors leading to social competence to be manifested (e.g., Cole, 2006). With ample variation in time lags we can measure effects of time continuously; this allows us to reach a better understanding of how effects manifest themselves over time, as we can disentangle direct from indirect pathways in which the various variables of interest contribute to social competence (Masten et al., 2006). Indeed, we are collecting longitudinal human data indexing social competence ranging from days (WP3: RADAR cohort) to yearly measurements (WP2: l-CID; WP3: Generation R, NTR, RADAR) to three-year intervals (WP1: YOUth cohorts) to generations (WP3: RADAR; TRAILS cohort). The RADAR cohort is of especial interest here as it is one of the few existing cohorts that even combines various lags within their data collection.

Then there are three remaining strands of research that according to Masten and Cicchetti (2010) are also instrumental in informing interventions, but which have not received as much attention in this paper. In all three cases, CID is able to contribute missing information. One line of research should be demonstrating the necessity of testing intervention designs that target mediating processes for change in social competence, which we cover in Work Package 2. A second strand of research should address how the interplay between genes, brain and environment affects social competence, which we address in our multi- method cohorts: YOUth-cohorts, l-CID and NTR all collect genes and multiple indices of environment (YOUth cohorts in WP1: Onland-Moret et al., 2020 twin cohorts L-CID in WP2; cf. Crone et al., 2020; NTR cohort in WP3: Boomsma et al., 2006; cf. Branje et al., 2020). We also have access to rodent models that allow for a level of control at the level of genes, brain, or environment that cannot be achieved in humans (WP4; cf., van der Veen et al., 2020). Finally, Masten and Cicchetti (2010) stress the need of well-designed experiments to further bolster our model that go beyond longitudinal cohorts to demonstrate causal directions between variables of interest and the outcome measure of social competence. With the help of animal models (WP4) and neurocognitive testing (WP1, WP2) we can achieve this. For instance, while Bierman et al. (2008) suggest that enriched environments foster social competence, we now test its specificity and generalizability of this in rodent models, which allows for hypothesis-testing in more stringent conditions as the contribution of other factors such as socio-emotional and genes are controlled for (van der Veen et al., 2020). All in all, the CID unites various strands of research that together centers on achieving a better understanding of the development of social competence.

## 6 Conclusions

To conclude, the literature is still missing a unified approach that integrates how a range of underlying skills together shapes the development of social competence in a range of contexts. The cohorts in CID collect information on different indices of social competence as well as on a wide range of underlying skills concerning the same children in a range of contexts repeatedly across various lags. The work packages in CID each provide unique additional information in testing our model. Putting these pieces together, CID aims to provide the evidence required for such theory-building and bridge these gaps in the literature.

## Declaration of Competing Interest

The authors declare that they have no known competing financial interests or personal relationships that could have appeared to influence the work reported in this paper.
