# Supplementary material for: The building blocks of social competence: Contributions of the Consortium of Individual Development
Source: Dev Cogn Neurosci. 2020 Sep 18;45:100861. doi: 10.1016/j.dcn.2020.100861 (PMC7509192; doi:10.1016/j.dcn.2020.100861)
Supplement: Supplementary file 1 [file mmc1.docx]

Supplementary Information

Table 1: Experimental tasks and questionnaires administered in five CID-cohorts targeting one of the skills underlying social competence. For a description of the tasks or questionnaires in each of the cohorts, see the individual references on the cohorts below.

| *Social*  *Competence skills* | CID COHORTS  Gen R L-CID RADAR TRAILS YOUth | | | | | |
| --- | --- | --- | --- | --- | --- | --- |
| social encoding | PCI ^BT {3}^ | PCI ^BT {3-7; 7-13}^ | ESCS ^BT {2}^  PCI ^BT {12-16}^ | ESCS ^BT {2.5}^  PCI ^{0.25; 2.5; 4.5}^  ANT Face Recognition  ^CT {11}^  ANT Identification of Facial Emotions ^CT {11}^  CSBQ ^Q {11; 13; 16}^ | Face-House  ^EEG {0-7} MRI {8-16}^  Face pop out  ^ET {0-7}^  PCI ^BT {0-7} {8-16}^  Emotion recognition (PENN)^CT {8-16}^  Face emotion  ^EEG {1-7} MRI {8-16}^  Social Gaze  ^ET {0-7} {8-16}^ |  |
| social problem solving | PCI ^BT {3; 10}^  Berkeley Puppet Interview ^BT {6}^  PEERS ^CT {7-8}^  Donating task ^BT {8}^  Prosocial Cyberball Task ^CT {8; 10}^  Friendship quality ^Q {10}^ | PCI ^BT {3-7; 7-13}^  Donating task ^BT {3-7; 7-13}^  Prosocial owl task ^CT {3-7}^  Prosocial Cyberball Task ^MRI {7-8; 9-10; 11-12} CT {8-9; 10-11; 12-13}^ | ESCS ^BT {2}^  PCI ^BT {2}^  Conflict Resolution Styles Inventory * ^Q {c: 12-27; p: 12-18; f: 12-18; i: 18-27}^ | ESCS ^BT {2.5}^  Erickson Scales  ^BT {2.5}^  Etch-a-Sketch  ^BT {4.5}^  ASQ ^Q {2.5}^  Prosocial behavior (ITSEA/SDQ) ^Q {2.5; 4.5)^  CBCL/YSR Social Problems scale ^Q {11; 13; 16}^ | PCI ^BT {0-7} {8-16}^  Trust game ^CT {8-16}^  Prosocial Cyberball Task ^CT {8-16}^ |  |
| emotion regulation | CAMPIS-SF ^BT {0.5; 1.2; 2}^  IBQ-R ^Q {0.5}^  PCI ^BT {1.2}^  Delay Gratification  ^BT {3}^  Inhibition ^Q {3; 13}^  CBQ-VSF ^Q {6}^  CEMS ^Q {6; 10}^  Inhibition  task ^BT {6; 8; 13}^  Lying/honesty task ^MRI {8}^  Gambling  task ^CT {10}^  Ultimatum  game ^CT {1}^ | Cheating task ^BT {3-4; 8-9} CT {7-8; 9-10}^  Delay Discounting ^CT {7-13}^  Delay Gratification ^BT {4-6; 7-10}^  CBQ/EATQ ^Q {3-7} {7-13}^  Social aggression task ^CT^ ^{8-9; 8-13} EEG {4-6} MRI {7-8; 7-12}^ | DERS ^q; {12-20}^  Daily mood device ^q; {12-221]^  Delay Gratification  ^BT {2}^ | Delay Gratification ^BT {2.5; 4.5}^  IBQ/ECBQ/CBQ  ^Q {0.25; 2.5; 4.5}^  PCI ^BT {2.5; 4.5}^  Etch-a-Sketch ^BT {4.5}^  EATQ ^Q {11, 16]^  SSRS ^Q {11}^ | Delay Discounting ^CT {8-16}^  Delay Gratification ^BT {2-7}^  IBQ/CBQ/EATQ  ^Q {0-7} {8-16}^  PCI ^BT {0-7} {8-16}^ |  |
| communi-cation | N-CDI ^CT {1.5}^  LDS ^Q {2.5}^  TVK ^Q {6}^  WISC-V – vocabulary ^BT {13}^ | PCI ^BT {3-7; 7-13}^ | ESCS ^BT {2}^  PCI ^BT {12-16}^ | ESCS ^BT {2.5}^  PCI ^BT {2.5; 4.5}^  ASQ ^Q {2.5)^  DB-DOS ^BT {4.5}^  EATQ ^Q {11, 16}^  Revised Class Play ^Q {11}^  SSRS ^Q {11}^  CSBQ ^Q {11; 13; 16}^ | LWL ^ET {2-4}^  N-CDI ^Q {1-4}^  PCI ^BT {0-7} {8-16}^  PPVT  ^CT {R3; R6; R9-R15}^  ASQ-SE^Q {0-4}^  CELF ^Q {2-7; 8-16}^ |  |
| empathy | My Child ^Q {6}^  ICU ^Q {10}^ | MyChild  ^Q {4-7} {7-13}^  Sally-Ann task ^BT {4-7}^  IRI *^Q {11;12}^*^1^ | IRI ^Q; {12-27}^  Empathy tasks  ^BT {16}^ | ITSEA ^Q {2.5)^  Prosocial Behavior  ^Q {11; 13; 16}^  CSBQ ^Q {11; 13; 16}^ | ToM battery ^BT{5-7}^  ITSEA ^Q {2-3}^ **  SDQ ^Q {2-7; 8-16}^  IRI ^Q {2-7; 8-16}^ |  |

*Notes:*

Cohorts: Gen R = Generation R (Kooijman et al., 2016); L-CID = Leiden Consortium on Individual Development (Crone et al., this issue); RADAR = Research on Adolescent Development and Relationships (Branje & Meeus, 2018; Crocetti, Branje, Rubini, Koot, & Meeus, 2017); TRAILS = Tracking Adolescents’ Individual Lives’ Survey (Ormel et al., 2012); YOUth = Youth of Utrecht (Onland-Moret et al., this issue).

BT = behavioral task; CT = Computer task; EEG = EEG task; ET = eye tracking task; MRI = task in MRI scanner; Q= questionnaire. PCI = parent-child interaction. * with different raters; ** Not all measures are available for the full cohort.

The values between {} refer to the ages sampled in years.

References:

Branje, S.J.T., & Meeus, W.H.J. (2018). Research on Adolescent Development and Relationships (young cohort). doi:10.17026/dans-zrb-v5wp

Crocetti, E., Branje, S., Rubini, M., Koot, H. M., & Meeus, W. (2017). Identity processes and parent–child and sibling relationships in adolescence: A five‐wave multi‐informant longitudinal study. Child Development, 88(1), 210-228.

Crone, E. A, Achterberg, M., Dobbelaar, S., Euser, S., van den Bulk, B., van der Meulen, M., van Drunen, L., Wierenga, L., Bakermans-Kranenburg, M.J., & van IJzendoorn, M.H. (this issue). Neural and behavioral signatures of social adaptation in childhood and adolescence: The Leiden Consortium on Individual Development (L-CID). *Developmental Cognitive Neuroscience*.

Kooijman, M. N., Kruithof, C. J., van Duijn, C. M., Duijts, L., Franco, O. H., van IJzendoorn, M. H., ... & Moll, H. A. (2016). The Generation R Study: design and cohort update 2017. *European Journal of Epidemiology, 31(12),* 1243-1264.

Onland-Moret, N.C., Buizer-Voskamp, J., Albers, M.E.W.A., Brouwer, R.M., Buimer, E.L., Hessels, R.S., de Heus, R., Huijding, J., Junge, C.M.M., Mandl, R.C.W., Pas, P., Vink, M., van der Wal, J.M., Hulshoff Poll, H.E., & Kemner, C (This issue). The YOUth study: Rationale, Design and Study Procedures. *Developmental Cognitive Neuroscience.*

Ormel, J., Oldehinkel, A. J., Sijtsema, J., van Oort, F., Raven, D., Veenstra, R., ... & Verhulst, F. C. (2012). The TRacking Adolescents' Individual Lives Survey (TRAILS): design, current status, and selected findings. *Journal of the American Academy of Child & Adolescent Psychiatry, 51(10)*, 1020-1036.
